# Evaluation of Plasma Soluble CD 40 Ligand (sCD40L) Levels as a Biomarker for Autism Spectrum Disorder: A Link to the Severity of Cognitive, Social, and Sensory Dysfunctions

**DOI:** 10.3390/jcm15218547

**Published:** 2026-11-03

**Authors:** Nadra Elyass Elamin, Dost Muhammad Halepoto, Mohamed Eid Soliman, Abdulrahman M. Alhowikan, Laila Yousef Al-Ayadhi

**Affiliations:** 1 Autism Research and Treatment Centre, Faculty of Medicine, King Saud University, Riyadh 11461, Saudi Arabia; 2 National Plan for Science and Technology, King Saud University, Riyadh 11461, Saudi Arabia; 3 Department of Physiology, Faculty of Medicine, King Saud University, Riyadh 11461, Saudi Arabia

**Keywords:** ASD, sCD40L, neuroinflammation, biomarker, inflammatory mediator, disease severity

## Abstract

**Background:** Soluble CD40 ligand (sCD40L) plays an important role in inflammation and autoimmune disorders, including a variety of neurological and neurodegenerative disorders. However, evidence regarding the role of sCD40L in autism spectrum disorder (ASD) is limited. Therefore, the aim of this study was to assess the clinical significance of plasma sCD40L levels in individuals with ASD and to examine their relationship with the severity of ASD, social impairments, and sensory dysfunction. **Materials and Methods:** Plasma levels of sCD40L were measured using an enzyme-linked immunosorbent assay (ELISA). The severity of ASD, social impairments, and sensory dysfunction was assessed in all children with ASD using the Childhood Autism Rating Scale (CARS), the Social Responsiveness Scale (SRS), and the Short Sensory Profile (SSP), respectively. **Results:** Plasma sCD40L levels were significantly increased in children with ASD (83.77 ± 24.76) compared with controls (68.28 ± 12.74), with *p* = 0.001. Plasma sCD40L levels were significantly higher in children with severe ASD-related symptoms. The mean sCD40L levels were 87.23 ± 20.93 among children with severe ASD according to the CARS, 86.45 ± 25.53 among those with greater social impairment according to the SRS, and 89.74 ± 27.22 among those with atypical sensory processing according to the SSP (*p* ≤ 0.05). These results indicate that sCD40L levels increased with greater severity of the symptoms. Additionally, receiver operating characteristic (ROC) curve analysis revealed significant discriminatory ability of plasma sCD40L between the two groups, with an AUC of 0.701 [95% confidence interval (CI), 0.583–0.819, *p* < 0.001]. **Conclusions:** Our study suggests that sCD40L may contribute to the pathophysiology of ASD and be associated with the severity of ASD symptoms. This association suggests that sCD40L may serve as a promising biomarker for neuroinflammatory processes associated with ASD. However, further studies with larger sample sizes are warranted to validate these findings.

## 1. Introduction

Autism spectrum disorder (ASD) is a diverse group of conditions characterized by difficulties in social communication and social interaction, along with restricted interests, repetitive patterns of behavior or activities, and sensory abnormalities [1]. Individuals with ASD exhibit substantial variability in their symptom severity and clinical manifestations [2]. The multifactorial etiology of ASD involves complex interactions between genetic, environmental, neurological, and immunological factors [3,4]. Individuals with ASD frequently present with multiple co-occurring conditions, including attention-deficit/hyperactivity disorder (ADHD), intellectual disability (ID), epilepsy, anxiety, gastrointestinal dysfunction, and mental health problems, which correlate with greater disease severity [1,5]. The prevalence of ASD has significantly increased in recent years. According to the most recent estimate from the Centers for Disease Control and Prevention (CDC), the prevalence of ASD has increased to 1 in 31 children in the United States [6]. Currently, the diagnosis of ASD depends on developmental history, clinical interviews, direct behavioral observations, and standardized behavioral assessments of the cognitive and developmental abilities of individuals with ASD [3,6]. Although several biomarkers have been investigated as potential diagnostic tools for ASD, no definitive biomarker has been validated for routine clinical application. Therefore, the development of early prediction and intervention strategies and the identification of diagnostic biomarkers to improve children’s outcomes are critically important.

Accumulating evidence suggests that immune dysregulation and neuroinflammation contribute to neurodegeneration and the pathophysiology of several neurological disorders, such as ASD, Alzheimer’s disease (AD), and Parkinson’s disease [7,8]. In ASD, alterations in innate immunity, microglial activity, inflammatory signaling, and cytokine and chemokine expression have been increasingly reported. These processes may impair synaptic development and neural circuitry, contributing to the behavioral and cognitive deficits observed in individuals with ASD [2,4,9,10].

CD40 ligand (CD40L) is a type II transmembrane glycoprotein that is a member of the tumor necrosis factor (TNF) superfamily. CD40L is widely expressed in activated T cells, B cells, epithelial cells, fibroblasts, endothelial cells (ECs), platelets, and microglia [11,12]. Upon cell activation, CD40L translocates to the cell surface as membrane CD40L (mCD40L), which is enzymatically cleaved to form soluble CD40L (sCD40L). mCD40L and sCD40L are biologically active [12]. Binding of sCD40L to its receptor CD40, a type I transmembrane glycoprotein expressed on various cell types, promotes receptor oligomerization and initiates downstream signaling pathways, thereby mediating its biological effects. sCD40L plays an important role as a mediator of inflammatory responses, promoting immune cell activation, expression of inflammatory adhesion molecules, leukocyte recruitment, tissue factor production, and coagulation cascade activation [13]. Furthermore, sCD40L may serve as a biomarker of immune dysregulation associated with autoimmune and autoinflammatory disorders [11].

The CD40/CD40L dyad plays a central role in the activation and regulation of humoral and cellular immune responses. Activation of this signaling pathway may promote the production of pro-inflammatory cytokines, increase oxidative stress, and disrupt synaptic function [7,8,9]. Within the central nervous system, CD40/CD40L signaling has been implicated in neuroinflammation by activating microglia and astrocytes and promoting inflammatory cascades, resulting in increased neuronal damage and impaired CNS homeostasis in neuroinflammatory disorders [11,12]. Therapeutic strategies targeting the CD40/sCD40L interaction may therefore help alleviate neuroinflammation and immune dysregulation, offering potential benefits for managing autoimmune and neuroinflammatory conditions.

sCD40L dysregulation has been reported in a wide range of inflammatory and immune-mediated conditions, including cardiovascular, neurological, neurodegenerative, and autoimmune diseases. Elevated sCD40L levels have been reported in patients with sepsis, rheumatic diseases, systemic lupus erythematosus, multiple sclerosis, chronic kidney disease, cardiovascular disease, and Alzheimer’s disease [11,12,13,14,15,16,17,18] and have been associated with the severity and progression of these diseases, underscoring sCD40L’s potential role as a biomarker and pathogenic mediator.

Despite the potential role of sCD40L in neurological disorders, neurodegenerative disorders, and neuroinflammatory processes, its role in ASD remains largely unexplored. The available evidence is limited to a single previous study [19], a longitudinal study of 104 children with ASD compared with 54 typically developing controls, measuring several immune mediators in blood and cerebrospinal fluid (CSF). The authors reported no significant differences in several blood inflammatory markers such as IL-6, TNF alpha, and IL-1beta between the two groups, while sCD40L and EGF were significantly increased in ASD subjects compared to the controls. Moreover, no changes were detected in CSF, with little correlation between peripheral blood and CSF immune profiles. These findings highlight the complexity of immune dysregulation in ASD and suggest distinct immunological mechanisms between the central nervous system compared with the peripheral circulation [19].

As the neurological basis of sCD40L in ASD is unclear, its contribution to the pathophysiology of ASD needs to be examined. This may help to evaluate its potential as an early diagnostic biomarker for ASD and its application in developing targeted therapeutics. Therefore, we aimed to explore the plasma levels of sCD40L in individuals with ASD compared with a control group; investigate the relationships between sCD40L and CARS, SRS, and SSP scores and age; and evaluate sCD40L’s diagnostic utility using ROC curve analysis.

## 2. Materials and Methods

### 2.1. Participants

Forty children with ASD aged 3–13 years (mean ± SD, 6.52 ± 2.61) and 40 age- and sex-matched controls aged 3–14 years (mean ± SD, 7.92 ± 3.74) were enrolled in this study. Children with ASD were recruited from the Autism Research and Treatment Center, Faculty of Medicine at King Khalid University Hospital Riyadh, King Saud University, Riyadh, Saudi Arabia. The Diagnostic and Statistical Manual of Mental Disorders (DSM)–IV was used to assess and evaluate all participants [1]. Clinical evaluations of children with ASD were performed based on their history, as obtained from parental interviews and clinical and neuropsychiatric assessments. Children with neurological disorders, any comorbid ASD-related disorders, metabolic disorders, inflammatory or infectious diseases, or any known medical conditions, as well as those who received any medication, were excluded from the study, as these conditions might have introduced additional challenges and biased the results.

The control group consisted of healthy children recruited from King Khaled University Hospital, the Healthy Child Outpatient Clinic of the Department of General Pediatrics, who attended routine follow-up. They were healthy children with no family history of neurological or psychiatric disorders and were not related to children with ASD.

This study was approved by the Institutional Review Board and Guidelines of Health Sciences Colleges Research on Human Subjects, College of Medicine, King Saud University, and was conducted in accordance with the Declaration of Helsinki (approval number: 25/0770/IRB). Written informed consent was obtained from the parents or legal guardians of all participants included in this study prior to their participation in the study.

### 2.2. ASD Behavioral Assessment

The severity of ASD, social impairments, and sensory performance was assessed in all children with ASD using the Childhood Autism Rating Scale (CARS), the Social Responsiveness Scale (SRS), and the Short Sensory Profile (SSP), respectively, as previously reported [20,21,22]. According to their total scores on the CARS and SRS, children with ASD were subclassified into mild-to-moderate and severe subgroups. For the CARS, scores between 30 and 36.5 indicate mild-to-moderate ASD, and scores between 37 and 60 indicate severe ASD [20]. For the SRS, scores of 60–75 indicate mild-to-moderate social impairment, while scores ≥ 76 indicate severe social impairment [21].

The total SSP score indicates overall sensory dysfunction; lower total SSP scores indicate greater sensory dysfunction. Accordingly, a score of 155–190 indicates typical performance, 142–154 probable sensory difficulties, and 38–141 definite sensory difficulties [22].

### 2.3. Blood Sample Collection and Measurement of Plasma sCD40L Levels

Blood samples, approximately 5 mL, were obtained from both groups after an overnight fast and placed in test tubes containing EDTA. All blood samples were centrifuged at 3000 rpm for 10 min at 4 °C. The obtained plasma was aliquoted and stored at −80 °C until further analysis.

Plasma sCD40L levels were measured using a commercially available enzyme-linked immunosorbent assay (ELISA) kit (Human sCD40L ELISA kit, ELK Biotechnology, Wuhan, China, cat. No. ELK 9196) according to the manufacturer’s instructions. The sCD40L concentration (pg/mL) was determined by comparing the optical density with a standard curve. All samples were measured in duplicate, and mean values are reported. The detection range of plasma sCD40L was 15.63–1000 pg/mL. The intra-assay and inter-assay coefficients of variation (CV%) of the ELISA kit were less than 8% and 10%, respectively. No significant cross-reactivity or interference was observed.

### 2.4. Statistical Analysis

Data were analyzed using IBM SPSS software, version 29.0 (IBM Inc., Armonk, NY, USA). The normality of the data was examined using the Shapiro–Wilk test. Continuous variables with normal distributions are presented as the mean ± standard deviation (SD) and were compared using an independent Student *t*-test. To explore the associations between plasma sCD40L levels and different behavioral scales (CARS, SRS, SSP) and age, Pearson’s correlation coefficient analysis was performed. Moreover, the diagnostic value of sCD40L in discriminating between the study groups was assessed using receiver operating characteristic (ROC) curve analysis, which is a useful method for evaluating the predictive power of biomarkers. It included calculations of the area under the curve (AUC) and its confidence interval (%CI), as well as the optimal threshold, sensitivity, and specificity. All data with *p*-values ≤ 0.05 were considered significant.

## 3. Results

Table 1 presents the general characteristics of the study participants in the ASD group (6.52 ± 2.61 years) and the age and sex-matched control group (7.92 ± 3.74). ASD subjects were stratified by the severity of their disease and social impairment, measured by CARS and SRS scores, respectively, into mild-to-moderate and severe subgroups. According to their SSP score, the ASD group was divided into three subgroups: the typical performance subgroup included those with an SSP score within the expected range of 155–190, the mild-to-moderate ASD subgroup included those with probable sensory difficulties (142–154), and the severe ASD subgroup included those with definite sensory difficulties (38–141), according to the conventional criteria described in Section 2. The mild-to-moderate and severe subgroups were combined into a single ASD subgroup (atypical subgroup) for subsequent analyses to facilitate comparison with the control group, and to ensure a more appropriate group size for statistical analysis. The results are presented as the mean ± SD. Results showed that 55% of ASD children had mild-to-moderate ASD, whereas 45% had severe ASD according to their CARS scores. In contrast, based on their SRS scores, a small percentage of the ASD children (32.5%) had mild-to-moderate ASD, and 67.5% had severe ASD. The overlap between the three scales was subsequently examined to determine the number of children with ASD who were simultaneously classified as having high severity on two or all three measures. Among them, 11 (27.5%) participants were classified as having severe behavioral symptoms on all three scales (CARS, SRS, and SSP). An additional five (12.5%) were classified as severe on the CARS and SRS, one (2.5%) on the CARS and SSP, and five (12.5%) on the SRS and SSP.

In this study, plasma sCD40L levels were significantly higher in individuals with ASD (83.77 ± 24.76) than in controls (68.28 ± 12.74) (*p* = 0.001). Furthermore, plasma sCD40L levels were compared between the ASD subgroups and controls. Table 1 shows that plasma sCD40L levels were increased in both CARS subgroups, but they were significantly increased only in the severe subgroup (87.23 ± 20.93) compared with the controls (68.28 ± 12.74, *p* = 0.001), suggesting their potential association with the severity of the disease. Similarly, based on SRS scores, children with severe ASD exhibited significantly higher plasma sCD40L levels (86.45 ± 25.53) compared with the controls (68.28 ± 12.74, *p* = 0.001), indicating that the progression of social impairment severity might be associated with increased plasma sCD40L levels. On the other hand, no significant difference in plasma sCD40L levels was observed in the mild-to-moderate subgroup (77.09 ± 21.89) compared with the severe ASD subgroup and controls (*p* ≥ 0.05).

Additionally, the ASD group was subdivided according to SSP scores, as an indicator of sensory impairment, into typical (normal performance) and atypical (including moderate and severe sensory difficulties) ASD subgroups. The mean plasma level of sCD40L tended to be higher in the atypical ASD subgroup (89.74 ± 27.22) than in the typical ASD subgroup (77.08 ± 20.23). Subgroup analysis showed that children in the atypical ASD subgroup (89.74 ± 27.22) had significantly higher sCD40L levels than the control group (68.28 ± 12.74, *p* = 0.003).

Additionally, children with ASD were subdivided into a subgroup aged six years or less and a subgroup older than six years. Significantly higher levels of sCD40L were observed in the ASD group of children older than six years, and in those aged six years or less, compared with the controls (*p* ≤ 0.01).

**Figure 1:**
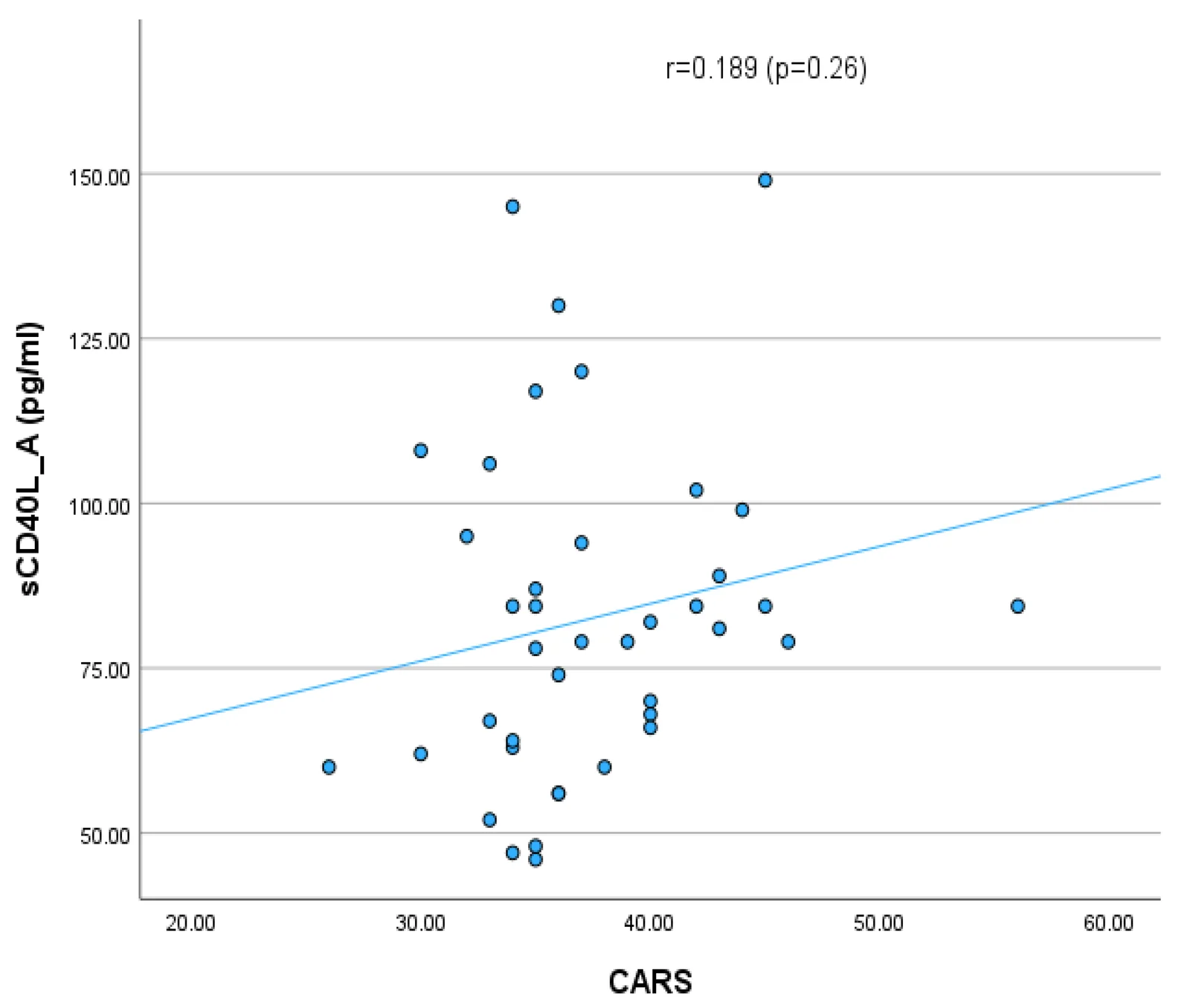
Pearson correlation coefficient (r) between sCD40L level and CARS score. Each blue circle represents an individual’s data point. The blue line represents the fitted linear regression line, showing the overall trend in the relationship between the variables.

**Figure 2:**
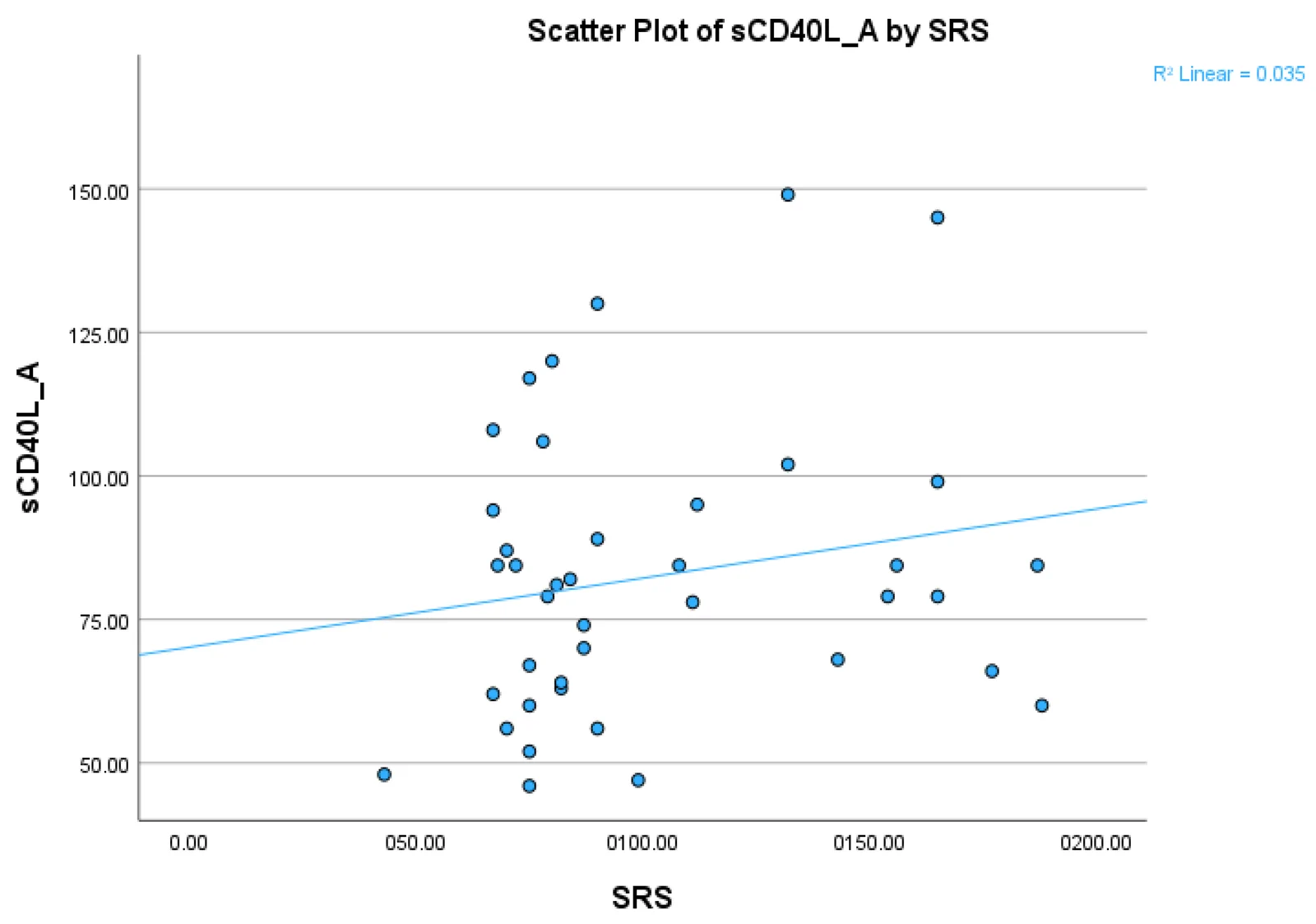
Pearson correlation coefficient (r) between sCD40L level and SRS score. Each blue circle represents an individual’s data point. The blue line represents the fitted linear regression line, showing the overall trend in the relationship between the variables.

**Figure 3:**
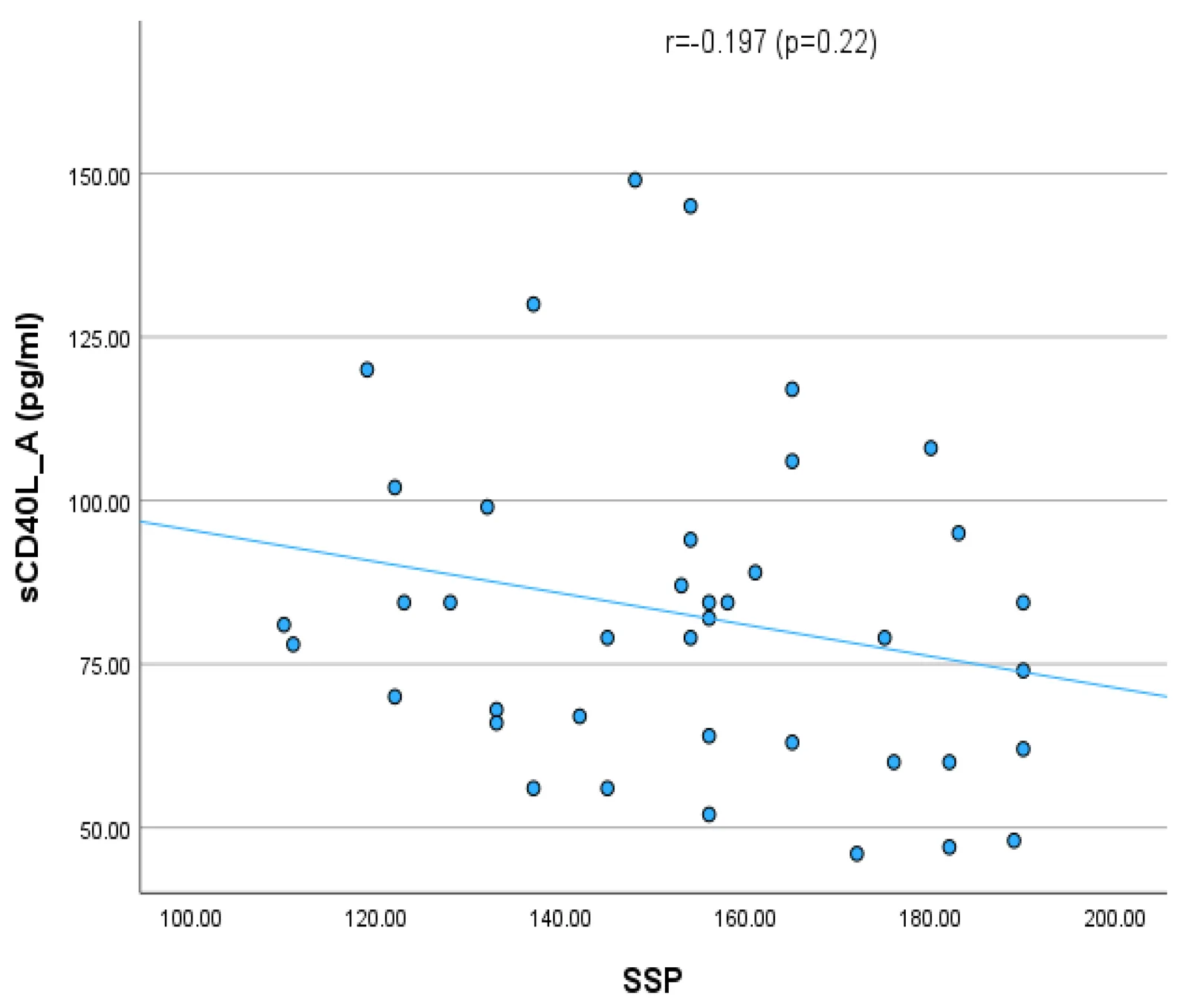
Pearson correlation coefficient (r) between sCD40L level and SSP score. Each blue circle represents an individual’s data point. The blue line represents the fitted linear regression line, showing the overall trend in the relationship between the variables.

**Figure 4:**
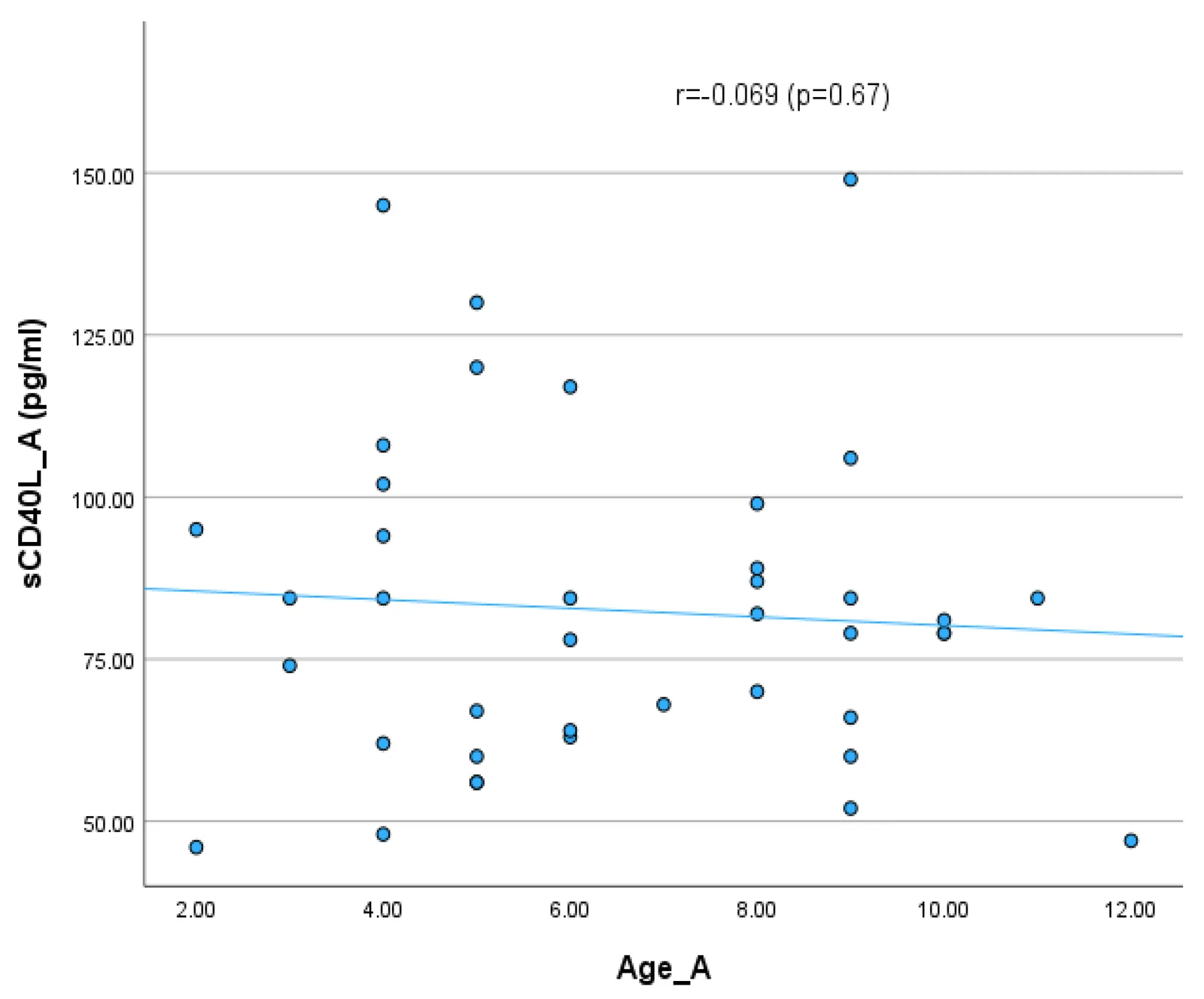
Pearson correlation coefficient (r) between sCD40L level and age. Each blue circle represents an individual’s data point. The blue line represents the fitted linear regression line, showing the overall trend in the relationship between the variables.

Furthermore, Pearson correlations were calculated to explore the possible correlation between plasma sCD40L levels and CARS, SRS, and SSP scores and age as measures of disease severity. However, our results showed no significant correlation between plasma sCD40L levels and CARS (r = 0.187, *p* = 0.25), SRS (r = 0.187, *p* = 0.25), and SSP scores (r = −0.197, *p* = 0.22) or age (r = −0.069, *p* = 0.67) (Figure 1, Figure 2, Figure 3 and Figure 4).

**Figure 5:**
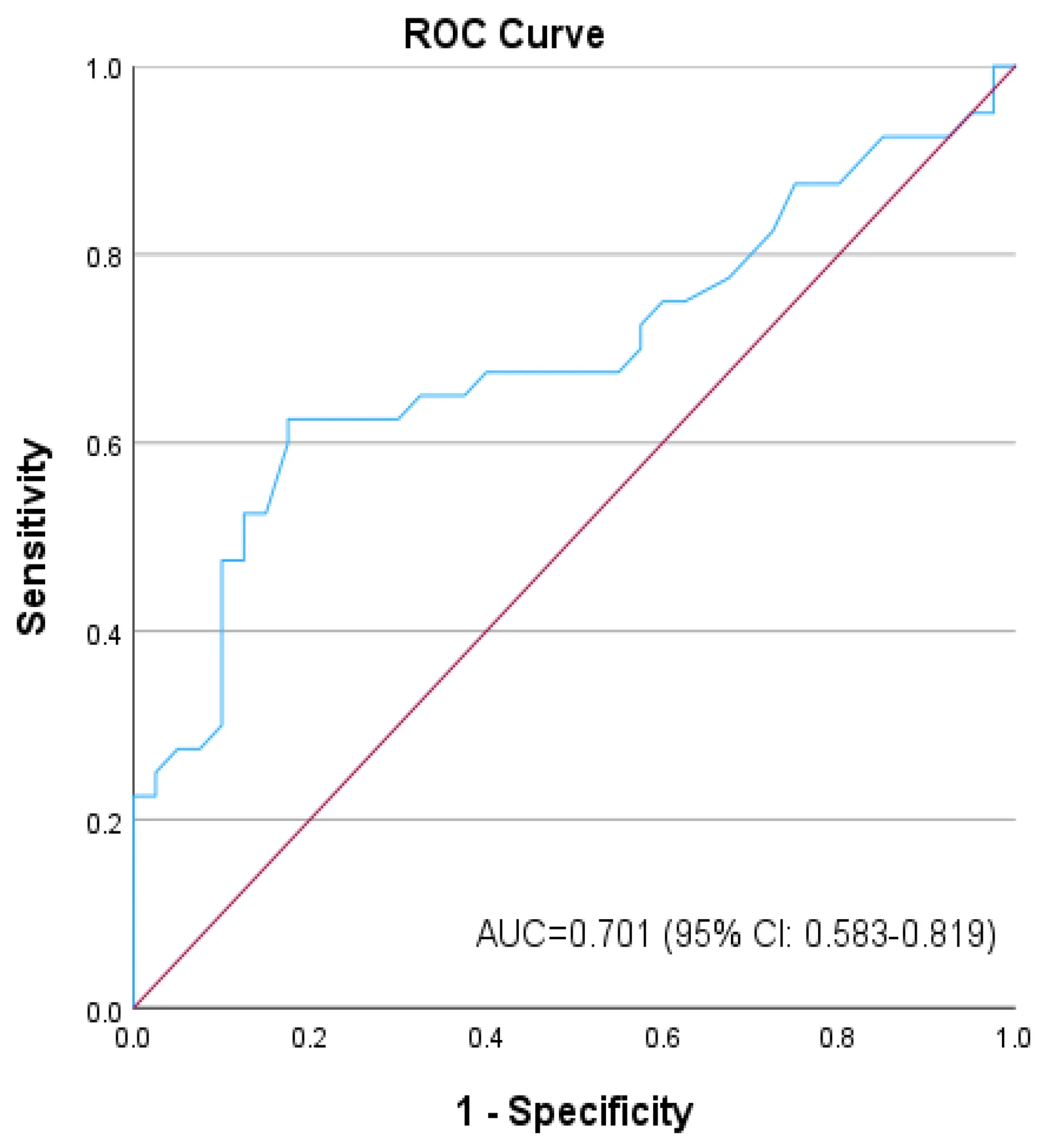
ROC curve analysis for plasma sCD40L in ASD group vs. control group. The blue curve represents the ROC curve, while the diagonal pink line indicates the reference line for no discriminatory ability.

An ROC curve analysis was conducted to evaluate the diagnostic ability of sCD40L to discriminate between the ASD and control groups. Table 2 and Figure 5 reveal that plasma sCD40L exhibited significant predictive ability to discriminate between the ASD and control groups (AUC = 0.701, 95% CI: 0.583–−0.819; *p* < 0.001) with 70% sensitivity and 60% specificity. Furthermore, we performed a separate analysis comparing the mild-to-moderate and severe subgroups with the control group. The ROC curve analysis did not demonstrate significant discriminatory performance between the mild ASD subgroup and the control group However, when ROC curve analysis was conducted using the severe subgroups, the discriminatory ability improved, and the sensitivity increased. sCD40L’s predictive value was greater in the CARS severe subgroup of ASD (AUC 0.812, *p* < 0.0001), moderate in the SRS severe subgroup of ASD (AUC 0.739, *p* = 0.001), and poor in the SSP atypical subgroup of ASD (AUC 0.624, *p* ≥ 0.05) compared with the controls (Table 3).

## 4. Discussion

Increasing evidence indicates that neuroinflammation is involved in the pathophysiology of ASD, leading to immune dysregulation and disruption of normal brain development, consequently influencing ASD-related behavioral features [23,24,25,26]. However, dysregulation of inflammatory mediators in the blood and cerebrospinal fluid has previously been reported in children with ASD and contributes to the severe core behavioral symptoms of ASD [23]. Among these inflammatory mediators, sCD40L is considered a key player in mediating inflammatory responses. Dysregulation of sCD40L has been implicated in a wide range of inflammatory and immune-mediated conditions and is increasingly associated with disease severity, underscoring its role as a biomarker and potential pathogenic mediator [8,12,14,18].

In the current study, plasma levels of sCD40L were significantly increased in children with ASD compared with controls (*p* = 0.001). Our findings are consistent with those of Pardo et al., 2017 [19], who reported an increase in serum sCD40L levels in children with ASD compared with controls and its implication in the pathogenesis of ASD [18]. Moreover, previous research suggests that sCD40L normally declines during development and may be elevated in psychiatric and neurodevelopmental conditions, with higher levels associated with lower cognitive and behavioral performance [18,19,27].

Given that sCD40L is intricately linked to neuroinflammation, cytokine signaling, and immune dysregulation, which are key factors in the pathogenesis of ASD, the elevated sCD40L level in the current study may reflect the inflammatory and immune abnormalities observed in individuals with ASD [2,24].

Individuals with ASD were stratified into mild-to-moderate and severe subgroups based on their CARS and SRS scores, and into typical and atypical performance subgroups based on their SSP scores. Both subgroups exhibited elevated plasma sCD40L levels compared with the controls, but the elevated levels in the mild-to-moderate subgroup did not reach statistical significance (*p* ≥ 0.05). Despite the lack of statistical significance, the observed upward trend may indicate a potential early biological process or signal that warrants further investigation. It is also possible that the lack of significance could be attributed to high inter-individual variability and sample size limitations. In contrast, the severe ASD subgroup exhibited significantly higher sCD40L levels compared with the controls, suggesting that sCD40L may be linked to the inflammatory and immune dysregulation associated with ASD symptoms severity as measured by the CARS, SRS, and SSP.

The increased sCD40L levels in the severe ASD subgroup in this study are consistent with previous studies documenting increased sCD40L levels and their contributing role in the pathogenesis of autoimmunity and inflammatory diseases, as well as their association with disease severity [14,15,16,28]. However, the prominent change in sCD40L suggests its potential as a biomarker associated with ASD symptoms severity. As sCD40L is known to play a contributing role in immune activation and neuroinflammation, its higher levels in severe cases may reflect increased inflammatory activity or immune dysregulation associated with disease severity [24]. However, this result should be interpreted with caution until further studies with larger sample sizes are conducted to confirm its validity.

The CD40/CD40L dyad plays a central role in the production of immune activation cytokines and the regulation of humoral and cellular immune responses [12]. In the central nervous system, CD40/CD40L signaling has been implicated in the neuroinflammatory response through its effects on microglia and astrocytes [29]. Upregulation of CD40 and sCD40L levels has previously been implicated in neurological and neurodevelopmental disorders and is associated with disease severity [30,31,32,33].

Aldosaari et al. [34] reported significantly increased numbers of CD40^+^ cells expressing pro-inflammatory mediators, including CCR1, IFN-g, TNF-a, and IL-17A, in children with ASD compared with a control group, suggesting altered inflammatory signaling associated with CD40+ immune cells in ASD. These inflammatory mediators play a vital role in disease progression [34]. Together with our finding of increased levels of sCD40L, these results suggest potential dysregulation of the CD40/sCD40L axis in ASD, warranting further investigation into its role in the pathophysiology of ASD and as a potential therapeutic target.

Notably, sCD40L levels were significantly elevated in children with ASD in both examined age groups compared with the controls (*p* > 0.05). Interestingly, sCD40L levels were higher in the subgroup of children aged six years or younger than in those older than six years, suggesting a potential age-related developmental pattern. This finding is consistent with a previous study in typically developing individuals, in whom sCD40L levels were reported to decrease across developmental stages, with higher levels observed in neonates and lower levels observed in adults [27]. This finding suggests that immune dysregulation may be more pronounced during early childhood and may decrease with age in a subset of children with ASD. Future longitudinal studies are warranted to determine whether the observed age-related decline is specific to ASD or reflects a broader developmental change.

Pearson correlation coefficient analysis was performed to assess the correlation between sCD40L and behavioral rating scale (CARS, SRS, SSP) scores and age. Although plasma sCD40L levels were significantly elevated in the severe ASD subgroup, our results revealed no significant correlation between plasma sCD40L levels and CARS, SRS, and SSP scores or age. The observed pattern may be attributable to other factors, including the complexity and heterogeneity of the ASD phenotype, age, and sample size, which may limit direct correlation. It is also possible that this finding reflects underlying biological processes, such as neuroinflammation, oxidative stress, immune activation, or other biological mechanisms, that may be associated with the severity of ASD [35]. This finding underscores the need for future studies with a larger sample size to further investigate this association.

To the best of our knowledge, this is the first study to evaluate the diagnostic utility of sCD40L in ASD using ROC curve analysis. We revealed that sCD40L showed a statistically significant moderate ability to discriminate between the study groups, as reflected by an AUC of 0.701 (95% CI 0.583–0.819). The 95%CI revealed a relatively wide range highlighting the high variability in sCD40L, which may be attributed to the limited sample size and the heterogeneity of sCD40L within the study population. This could reduce its statistical power.

Furthermore, ROC curve analysis of the severe ASD subgroup demonstrated improved discriminatory ability and increased sensitivity compared with the controls. sCD40L’s predictive value was greater in case of the CARS severe subgroup of ASD (AUC 0.812, *p* < 0.0001), moderate in the SRS severe subgroup of ASD (AUC 0.739, *p* = 0.001), and poor in the SSP atypical subgroup of ASD (AUC 0.624, *p* ≥ 0.05), compared with the controls, suggesting that sCD40L may have greater discriminatory potential in children with severe ASD symptoms, and that the performance of sCD40L may depend on the clinical characteristics and severity of the symptoms within the examined ASD subgroup. These results confirmed the observed elevated levels of sCD40L in the severe ASD subgroups compared with the controls and highlighted sCD40L’s potential as a biomarker of neuroinflammation associated with disease severity.

However, these preliminary results should be interpreted with caution. The relatively small sample size, cross-sectional design, and use of a single measurement of peripheral blood sCD40L are important limitations of the present study. Moreover, peripheral sCD40L levels may not fully reflect immune activity in the central nervous system. Further studies with larger sample sizes, longitudinal designs, and repeated measurements are therefore needed to confirm these findings and better understand the potential clinical relevance of sCD40L in ASD. Additionally, combining sCD40L with other biomarkers is essential to improve diagnostic accuracy and confirm the generalizability of the results [36].

## 5. Conclusions

Our findings indicate that elevated plasma sCD40L levels in children with ASD may be associated with the pathophysiology of ASD. Furthermore, elevated levels of sCD40L in the severe ASD subgroup may provide further insight into the potential involvement of inflammatory and immune-related mechanisms in ASD pathophysiology. These findings also suggest that sCD40L may be associated with ASD and may have discriminatory potential, particularly in severe ASD. Larger, well-characterized and longitudinal studies are warranted to validate these findings; clarify the relationships between sCD40L, neuroinflammation, and ASD severity; and determine sCD40L’s therapeutic potential in clinical settings.

## Figures and Tables

**Table 1:** General characteristics, and plasma sCD40L levels in ASD and control groups, and their associations with CARS, SRS, and SSP scores and age.

Variable	Group	N (%)	Mean ± SD	Relative Change from Control (%)	*p*-Value	Score
	Control	40	68.28 ± 12.74	100		
	ASD	40	83.77 ± 24.76	122.7	0.001 *	
**CARS**	Mild-to-moderate	22 (55%)	80.28 ± 27.21	117.6	0.06 **	≤36.5
	Severe	18 (45%)	87.23 ± 20.93	127.8	0.001 ***	>36.5
**SRS**	Mild-to-moderate	13 (32.5%)	77.09 ± 21.89	112.9	0.18 **	≤75
	Severe	27 (67.5%)	86.45 ± 25.53	126.6	0.001 ***	≥76
**SSP**	Typical performance	20 (50%)	77.08 ± 20.23	112.9	0.08 ^a^	≥155
	Atypical performance	20 (50%)	89.74 ± 27.22	131.4	0.003 ^b^	38–154
**Age**	≤6 years	22 (55%)	85.21 ± 26.41	124.8	0.009 ^$^	
	>6 years	18 (45%)	81.21 ± 22.57	118.9	0.007 ^$$^	

* ASD children vs. control subjects; ** mild-to-moderate vs. control subjects; *** severe vs. control subjects; ^a^ typical SSP vs. control subjects; ^b^ atypical SSP vs. control subjects; ^$^ comparison between ≤6 years old subgroup of autistic subjects and control subjects; ^$$^ comparison between >6 years old subgroup of autistic subjects and control subjects. A *p*-value ≤ 0.05 was considered statistically significant.

**Table 2:** ROC curve for plasma sCD40L in ASD group vs. control group.

Biomarker	AUC	Cutoff Value	Sensitivity	Specificity	*p*-Value	95% CI
sCD40L	0.701	69.5	70%	60%	<0.001	0.583–0.819

ROC, receiver operating characteristic; AUC, area under curve; CI, confidence interval.

**Table 3:** ROC curve analysis for plasma sCD40L in severe ASD subgroups according to CARS, SRS, and SSP vs. control group.

Biomarker	AUC	Cutoff Value	Sensitivity %	Specificity %	*p*-Value	95% CI
**CARS**	0.812	78	77.8	82.5	0.0001	0.690–0.933
**SRS**	0.739	77.5	66.7	82.5	0.001	0.612–0.866
**SSP**	0.624	78	55	82	0.121	0.453–0.795

ROC, receiver operating characteristic; AUC, area under curve; CI, confidence interval; CARS, Childhood Autism Rating Scale; SRS, Social Responsiveness Scale; SSP, Short Sensory Profile.

## Data Availability

All data generated or analyzed during this study are available from the corresponding author upon request.

## Abbreviations

ASD	Autism spectrum disorder
ADHD	Attention-deficit/hyperactivity disorder
AUC	Area under the curve
CARS	Childhood Autism Rating Scale
CDC	Center for Disease Control and Prevention
DSM-5	Statistical Manual of Mental Disorders 5
ID	Intellectual disability
ROC	Receiver operating characteristic
sCD40L	Soluble CD 40 ligand
SRS	Social Responsiveness Scale
SSP	Short Sensory Profile
TNF	Tumor necrosis factor superfamily

## References

[1] American Psychiatric Association (2013). Diagnostic and Statistical Manual of Mental Disorders: DSM-5™.

[2] Xu M., Zhang X., Liu Y., Yang Y., Zhou Z., Ma J., Shen S. (2026). Mesenchymal stem/stromal cell-based therapies for autism spectrum disorder: Emerging evidence and clinical prospects. J. Transl. Med..

[3] Khachadourian V., Mahjani B., Sandin S., Kolevzon A., Buxbaum J.D., Reichenberg A., Janecka M. (2023). Comorbidities in autism spectrum disorder and their etiologies. Transl. Psychiatry.

[4] Lv T., Wang M., Kui L., Wu J., Xiao Y. (2025). Novel inflammatory biomarkers for autism spectrum disorder detected by plasma Olink Proteomics. Children.

[5] Wang L., Wang B., Wu C., Wang J., Sun M. (2023). Autism Spectrum Disorder: Neurodevelopmental Risk Factors, Biological Mechanism, and Precision Therapy. Int. J. Mol. Sci..

[6] Shaw K.A., Williams S., Patrick M.E., Valencia-Prado M., Durkin M.S., Howerton E.M., Ladd-Acosta C.M., Pas E.T., Bakian A.V., Bartholomew P. (2025). Prevalence and Early Identification of Autism Spectrum Disorder Among Children Aged 4 and 8 Years—Autism and developmental disabilities monitoring network, 16 sites, United States, 2022. MMWR Surveill. Summ..

[7] Runge K., Fiebich B.L., Kuzior H., Rausch J., Maier S.J., Dersch R., Nickel K., Domschke K., van Elst L.T., Endres D. (2023). Altered cytokine levels in the cerebrospinal fluid of adult patients with autism spectrum disorder. J. Psychiatr. Res..

[8] Imbriani P., D’Ambra C., De Mori R., Ionta M., Renna A., Bonsi P. (2026). Dysregulation of Immune Mediators and Synaptic Plasticity in Central Nervous System Disorders. Cells.

[9] Than U.T.T., Nguyen L.T., Nguyen P.H., Nguyen X.H., Trinh D.P., Hoang D.H., Nguyen P.A.T., Dang V.D. (2023). Inflammatory mediators drive neuroinflammation in autism spectrum disorder and cerebral palsy. Sci. Rep..

[10] Noori A.S., Rajabi P., Sargolzaei J., Alaghmand A. (2024). Correlation of biochemical markers and inflammatory cytokines in autism spectrum disorder (ASD). BMC Pediatr..

[11] Liang Y., Zhu C., Sun Y., Li Z., Wang L., Liu Y., Li X., Ma X. (2021). Persistently higher serum sCD40L levels are associated with outcome in septic patients. BMC Anesthesiol..

[12] Zinellu A., Mangoni A.A. (2025). sCD40 and sCD40L as candidate biomarkers of rheumatic diseases: A systematic review and meta-analysis with meta-regression. Front. Immunol..

[13] Ozde S., Mehdizade C., Ali O.M., Ozde C., Kayapinar O., Erguven M. (2025). Evaluation of Plasma Soluble CD40 Ligand Levels in Children with Familial Mediterranean Fever and Its Relationship with Carotid Intima-Media Thickness. Diagnostics.

[14] Kim K.J., Baek I.W., Yoon C.H., Kim W.U., Cho C.S. (2017). Elevated levels of soluble CD40 ligand are associated with antiphospholipid antibodies in patients with systemic lupus erythematosus. Clin. Exp. Rheumatol..

[15] Xie J.X., Alderson H., Ritchie J., Kalra P.A., Xie Y., Ren K., Nguyen H., Chen T., Brewster P., Gupta R. (2017). Circulating CD40 and sCD40L Predict Changes in Renal Function in Subjects with Chronic Kidney Disease. Sci. Rep..

[16] Neamah M.Z., Sharquie I.K., AL Gawwam G. (2025). Serum Interleukin-40 and Soluble CD40 Ligand as complementary biomarkers for disease activity in multiple sclerosis patients. Medeni. Med. J..

[17] Shami A., Edsfeldt A., Bengtsson E., Nilsson J., Shore A.C., Natali A., Khan F., Lutgens E., Gonçalves I. (2021). Soluble CD40 Levels in Plasma Are Associated with Cardiovascular Disease and in Carotid Plaques with a Vulnerable Phenotype. J. Stroke.

[18] Desideri G., Cipollone F., Necozione S., Marini C., Lechiara M.C., Taglieri G., Zuliani G., Fellin R., Mezzetti A., di Orio F. (2008). Enhanced soluble cd40 ligand and alzheimer’s disease: Evidence of a possible pathogenetic role. Neurobiol. Aging.

[19] Pardo C.A., Farmer C.A., Thurm A., Shebl F.M., Ilieva J., Kalra S., Swedo S. (2017). Serum and cerebrospinal fluid immune mediators in children with autistic disorder: A longitudinal study. Mol. Autism.

[20] Schopler E., Van Bourgondien M.E., Wellman G.J., Love S.R. (2010). Childhood Autism Rating Scale (CARS2).

[21] Constantino J.N., Davis S.A., Todd R.D., Schindler M.K., Gross M.M., Brophy S.L., Metzger L.M., Shoushtari C.S., Splinter R., Reich W. (2003). Validation of a brief quantitative measure of autistic traits: Comparison of the social responsiveness scale with the autism diagnostic interview-revised. J. Autism Dev. Disord..

[22] Williams Z.J., Failla M.D., Gotham K.O., Woynaroski T.G., Cascio C. (2018). Psychometric Evaluation of the Short Sensory Profile in Youth with Autism Spectrum Disorder. J. Autism Dev. Disord..

[23] Liao X., Yang J., Wang H., Li Y. (2020). Microglia mediated neuroinflammation in autism spectrum disorder. J. Psychiatr. Res..

[24] Mostafa G.A., ElGebaly H.H., Shehab A.A., Mohamed M.A. (2022). Up-regulated serum levels of TAM receptor tyrosine kinases in a group of Egyptian autistic children. J. Neuroimmunol..

[25] Chen Y., Du X., Zhang X., Li F., Yuan S., Wang W., Zhu Z., Wang M., Gu C. (2025). Research trends of inflammation in autism spectrum disorders: A bibliometric analysis. Front. Immunol..

[26] Ferencova N., Visnovcova Z., Ondrejka I., Hrtanek I., Bujnakova I., Kovacova V., Macejova A., Tonhajzerova I. (2023). Peripheral Inflammatory Markers in Autism Spectrum Disorder and Attention Deficit/Hyperactivity Disorder at Adolescent Age. Int. J. Mol. Sci..

[27] Cholette J.M., Blumberg N., Phipps R.P., McDermott M.P., Gettings K.F., Lerner N.B. (2008). Developmental changes in soluble CD40 ligand. J. Pediatr..

[28] Pazoki A., Dadfar S., Shadab A., Haghmorad D., Oksenych V. (2024). Soluble CD40 Ligand as a Promising Biomarker in Cancer Diagnosis. Cells.

[29] Ots H.D., Tracz J.A., Vinokuroff K.E., Musto A.E. (2022). CD40–CD40L in Neurological Disease. Int. J. Mol. Sci..

[30] Wang M.T., Zhao R.C., Huang M., Chen J., Li X.P., Liu Y.H., Wang Y.J. (2026). Plasma profiling of B lymphocyte-associated cytokines in Alzheimer’s disease. J. Alzheimers Dis..

[31] Masuda H., Mori M., Umehara K., Furihata T., Uchida T., Uzawa A., Kuwabara S. (2018). Soluble CD40 ligand disrupts the blood–brain barrier and exacerbates inflammation in experimental autoimmune encephalomyelitis. J. Neuroimmunol..

[32] Giunta B., Rezai-Zadeh K., Tan J. (2010). Impact of the CD40-CD40L dyad in Alzheimer’s disease. CNS Neurol. Disord. Drug Targets.

[33] Wu Q., Wang Q., Yang J., Martens J.W., Mills E.A., Saad A., Chilukuri P., A Dowling C., Mao-Draayer Y. (2021). Elevated sCD40L in Secondary Progressive Multiple Sclerosis in Comparison to Non-progressive Benign and Relapsing Remitting Multiple Sclerosis. J. Cent. Nerv. Syst. Dis..

[34] Aldossari A.A., Ansari M.A., Nadeem A., Attia S.M., Bakheet S.A., Al-Ayadhi L.Y., Alanazi M.M., Shahid M., Alwetaid M.Y., Hussein M.H. (2023). Upregulation of Inflammatory Mediators in Peripheral Blood CD40+ Cells in Children with Autism Spectrum Disorder. Int. J. Mol. Sci..

[35] Hughes H.K., Moreno R.J., Ashwood P. (2023). Innate immune dysfunction and neuroinflammation in autism spectrum disorder (ASD). Brain Behav. Immun..

[36] El-Ansary A., Al-Ayadhi L.Y., Alfawaz H.A., Al-Ghabban H., Bjørklund G. (2026). Plasma KCC2, NKCC1, and GABA as peripheral biomarkers in autism spectrum disorder: A combined ROC analysis. Metab. Brain Dis..

